# Prevalence and predictors of asymptomatic malaria infection in Boricha District, Sidama Region, Ethiopia: implications for elimination strategies

**DOI:** 10.1186/s12936-023-04722-z

**Published:** 2023-09-26

**Authors:** Desalegn Dabaro, Zewdie Birhanu, Wondimagegn Adissu, Daniel Yilma, Delenasaw Yewhalaw

**Affiliations:** 1 Yirgalem Hospital Medical College, Yirgalem, Ethiopia; 2 Tropical and Infectious Diseases Research Center, Jimma University, Jimma, Ethiopia; 3 Department of Health Education and Behavioral Sciences, Institute of Health, Jimma University, Jimma, Ethiopia; 4 School of Medical Laboratory Sciences, Institute of Health, Jimma University, Jimma, Ethiopia; 5 Department of Internal Medicine, Institute of Health, Jimma University, Jimma, Ethiopia; 6 Clinical Trial Unit, Jimma University, Jimma, Ethiopia

**Keywords:** Prevalence, *Plasmodium* species, Risk factors, Asymptomatic malaria, Ethiopia

## Abstract

**Background:**

Malaria remains a major public health threat in Ethiopia despite the tremendous progress made towards the 2030 elimination targets. The silent transmission of asymptomatic infection is one of the factors that enhance the persistence of the disease as a public health issue and impedes efforts to eliminate malaria. Thus, this study aimed at investigating the prevalence and risk factors of asymptomatic malaria infection in Boricha district, Sidama region of Ethiopia.

**Methods:**

A community-based cross-sectional study was conducted in eight selected kebeles (smallest administrative unit) in Boricha district. Representative households were chosen using a multi-stage sampling technique. A total of 573 participants were included in the study. Malaria diagnosis was performed using rapid diagnostic test (RDT) and microscopy. A structured questionnaire was administered to collect socio-demographic information. Epi data 3.1 was employed for data entry, and SPSS version 25 was used for analysis.

**Results:**

Of the 573 asymptomatic participants tested, 6.1% were found to be positive by RDT and 4.0% by microscopy. Participants aged under 5 years (AOR = 1.57, 95% CI 0.46–5.39) and 5–14 years old (AOR = 2.42, 95% CI 1.08–5.40), Insecticide-treated net utilization (AOR = 8.41; 95% CI 1.09–65.08), travel history (AOR = 6.85, 95% CI 2.32–20.26) and living in a house with windows (AOR = 2.11, 95% CI 1.02–4.36) were significantly associated with the asymptomatic malaria infection.

**Conclusion:**

The findings of this study revealed that prevalence of asymptomatic malaria infection was higher in the study area. As a result, rigorous implementation of existing interventions, such as vector control and anti-malaria drugs, is strongly recommended. In addition, devising new ones that are suited to the contextual situations is highly suggested.

## Background

Malaria elimination is a global programme aiming to eliminate the disease by 2030. Countries worldwide have embraced this ambitious plan and have been working towards its realization. Concerning this, the World Health Organization (WHO) has recommended the optimal utilization of existing interventions while promoting research and innovation for the development of novel tools. As a result, tailored interventions that take into account local conditions have been implemented, with a strong emphasis on vector control, early diagnosis, and timely treatment [1, 2].

In relation to this, the WHO has developed the global technical strategy (GTS) for malaria 2016–2030, with clear targets for 2030 and specific milestones to measure progress in 2020 and 2025 [1, 2]. However, the 2020 GTS milestones were not achieved globally, as the incidence rate exceeded the projected target and there was an increase in malaria-related deaths as well. Similarly, the WHO African region, which accounts for a substantial share (95%) of global malaria cases and deaths, fell short of the GTS 2020 targets, except for a few countries [3].

Ethiopia has emerged as one of the few countries in the region that successfully met the GTS 2020 targets, leading to a notable 40% reduction in both malaria mortality and morbidity rates [3]. Consecutive Malaria Indicator Surveys conducted in 2007 and 2015 further emphasized the country’s efforts to control malaria, revealing a reduction in prevalence from 0.9 to 0.5%. Furthermore, the Health Management Information System indicated a persistent decline in confirmed malaria cases, demonstrating a 31% reduction between 2016 and 2020 [4]. Overall, between 2015 and 2019, there was a significant reduction in malaria incidence, falling from 5.2 million to below 1 million cases, accompanied by a decline in malaria-related deaths from 3.6 to 0.3 per 100,000 individuals at risk [5].

Despite remarkable progress mentioned earlier, the country has encountered several challenges toward malaria elimination. These include insecticide and drug resistance, changes in vector behaviour, the presence of infection reservoirs, and low healthcare-seeking behaviour [6–9]. The impact of COVID-19 pandemic on the healthcare system [7, 10, 11], and the spread of a new malaria vector, *Anopheles stephensi*, further threaten the elimination efforts [12, 13].

Despite the challenges encountered, there is still potential to achieve the elimination goal by sustaining the progress made so far and intensifying the use of existing interventions [9]. In this regard, early case detection and prompt treatment is highly important to halt and reverse the transmission. Thus, the national and global malaria elimination framework recommends a comprehensive approach that includes passive, active, and reactive case detection activities, along with effective treatment, to ultimately achieve the goal of elimination [1, 2, 14].

However, most often malaria diagnosis has predominantly relied on passive case detection, limiting the ability to track elimination progress [3, 4]. In addition, national and global surveillance data primarily come from healthcare sectors and focus on symptomatic patients, overlooking a significant portion of asymptomatic infections in apparently healthy individuals who can still spread the disease [15–18]. Hence, conducting comprehensive investigations of both symptomatic and asymptomatic malaria cases is vital for evidence-based planning, monitoring, evaluation, and identifying intervention gaps for reasonable improvement [1, 2, 9, 19].

In Ethiopia, there have been few studies highlighting the widespread presence of asymptomatic malaria infection [20–26]. However, there was a lack of studies conducted in the Sidama regional state of Ethiopia that specifically addressed this particular issue.

The aim of this study was to investigate the prevalence and associated factors of asymptomatic malaria infection in the Boricha district, one of the malaria-endemic districts in the region.

## Methods

### Study setting

The study was conducted in the Boricha district in Sidama regional state of Ethiopia. It lies at an average elevation of 1001 to 2076 m above sea level. The average annual temperature is between 17.6 and 22.5 °C, while the average annual rainfall is between 801 and 1000 mm [27, 28]. The district has a total population of 325,161 and consists of 42 kebeles (the smallest administrative unit), 39 rural, and 3 urban [29]. The majority of people live in rural areas where agriculture is the main source of livelihood. Coffee, maize, sugarcane, inset/false banana (Scientific name) and khat (*Khata edulis*) are the common crops cultivated in the area as well [28].

**Fig. 1:**
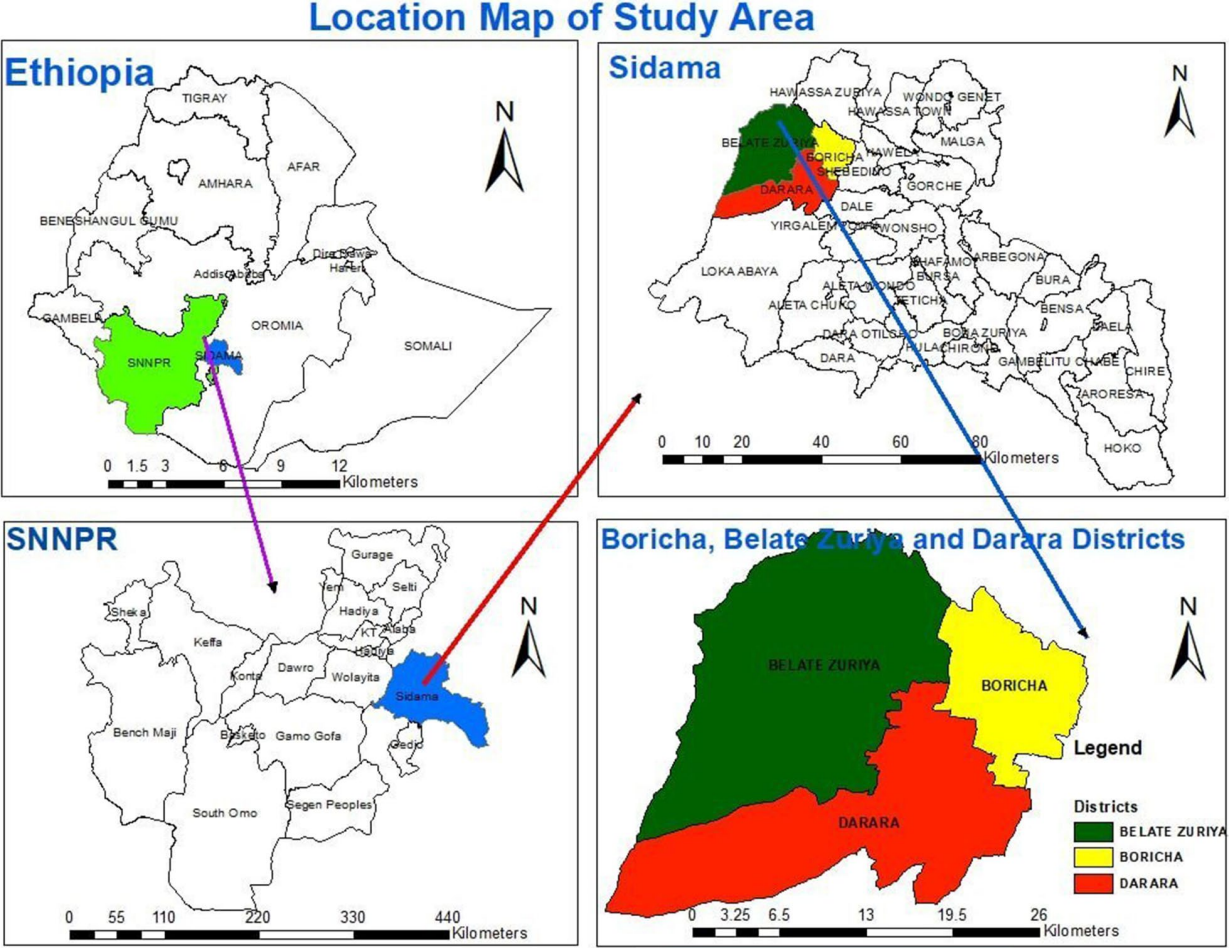
Map of the study area

To provide further information about the study setting, it is important to mention that the Boricha district was later divided into three separate districts: Boricha, Bilate Zuria, and Darara (Fig. 1). However, since the current study was a part of research project that had initiated prior to this administrative change, we focused on the kebeles that were originally recognized as part of Boricha before its disintegration. Prior to the division, Boricha comprised 42 kebeles (39 rural and 3 urban) with a population of 325,161. Following the division, Boricha was restructured into 14 kebeles (13 rural and 1 urban) with a population of 124,465. Similarly, Bilate Zuria consists of 18 kebeles (17 rural and 1 urban) with a population of 140,642, while Darara has 15 kebeles (14 rural and 1 urban) with a population of 112,037 [30].

Malaria is one of the most common infectious diseases in this district. In the area, *Plasmodium falciparum* and *Plasmodium vivax* are the most frequent species causing disease, according to the prior study. Both species are present throughout the year, with September to December being the greatest transmission season [31].

In the study setting, there has recently been a concerning increase in malaria cases. The total number of malaria cases rose from 6461 to 11,474. Specifically, in Boricha, the number of cases increased from 5177 to 6030, in Bilate Zuria from 902 to 4267, and in Darara from 382 to 1177 [30].

Thus far, the district, like other regions within the country, has been actively engaged in implementing a range of interventions to combat malaria. These interventions primarily focus on preventing the transmission of malaria-causing parasites and the mosquitoes responsible for spreading the disease. Key preventive measures include early detection and prompt treatment of malaria cases, along with the distribution of insecticide-treated bed nets (ITNs) and indoor residual spraying (IRS) [30].

Moreover, in selected urban areas, environmental management techniques have been employed to eliminate mosquito breeding sites within the community, thereby enhancing the effectiveness of prevention strategies. In addition, the region and district have been applying larvicides on mosquito breeding sites as a chemoprevention intervention. However, there is a lack of well-documented records regarding the coverage of this intervention.

As per the regional health bureau, the study area had successfully achieved complete coverage of ITNs before 2018 [30]. However, during the study period in 2021, the distribution of bed nets did not happen in the district, even though three years had passed since it was originally scheduled to occur.

### Study design

A cross-sectional study design employed to assess the prevalence of asymptomatic malaria infection, from April and May 2021. The survey took place at the household level, where individuals of any age and sex participated.

### Sample size determination

The sample size of the study was determined using a single population formula, n = (Z_a/2_^2^p (1 − p))/d^2^, assuming 21.8% prevalence of malaria in the field from previous study [31], with the 5% of margin of error, 95% of the confidence interval with the design effect of 2 and 10% of non-response rate. With the assumptions mentioned above, the final sample size was determined to be 576.

### Sampling techniques

A multi-stage sampling technique was used to select study participants. The district has a total of 42 kebeles, 39 rural and 3 urban. During the study period, some kebeles were difficult to access due to transportation limitations, while security concerns added to the challenges in other areas. Therefore, the study was limited to only eight kebeles due to constraints in time and financial resources. These kebeles were selected based on their accessibility and feasibility. Seven of them (Sadamo Chala, Harira Badalicha, Worancha Wacho, Konsore Chafe, Fulasa Aldada, Korangoge, Shondolo Liwo) were rural and one (Balela) was urban.

Each kebele is further divided into gote, locally used terms for small villages within the kebele. Each gote or small village was once more divided into the health development army (HDA), a number of households grouped for the easy provision of health education. The data of gote and HDA were obtained from the health posts in each kebele. The gotes within the kebeles were then initially selected at random. Following the selection of gotes, the HDA was then chosen randomly. Then, each HDA that was chosen was taken into account as a cluster of data collection. The health extension workers directed the selection of gotes and later HDAs. Then, a household was selected at random from each cluster. The feasibility of gote within the selected kebeles was also taken into consideration during data collection.

After providing sufficient information about the study, one volunteer was finally selected from among the chosen household members. During the interview a head of households or representatives of it provide information. The individuals who took part in this study appeared to be healthy, showing no symptoms or signs of malaria. In order to minimize the influence of previous malaria infections on the current study results individuals diagnosed with and treated for malaria at least within the last 3 months were excluded.

### Data quality management

Three medical laboratory technologists, two data collectors and one supervisor were employed for data collection. All of them received appropriate training and competency assessment ahead of data collection in order to ensure the quality of data. The contents of training included the data collection tools, variables of interest, the objective, rationale and significance of the study.

### Blood sample collection

Informed consent was sought from people invited to participate in the study. Then finger-prick blood was taken from people who gave their consent to participate in the study. Malaria was examined using the conventional rapid diagnostic test (cRDT) (SD Bioline Malaria Ag *P. falciparum* and *P. vivax*) and microscopy. After an on-site RDT examination, thin and thick blood films (BF) were prepared for microscopic examinations in the laboratory. Following proper preparation and getting dried, only thin BF was fixed by carefully dropping methanol. Then, both thick and thin BF was stained with 10% Giemsa. A light microscope with an oil immersion objective was used to examine the stained slides (100×) [32]. Malaria-positive participants were treated in accordance with the national treatment guideline of Ethiopia [33].

### Data analysis

The data was entered using Epi-Data 3.1, and the analysis was performed using SPSS version 25. Descriptive statistics were used to summarize the frequency distribution of the variables, and bivariate and multivariate logistic regression analysis was used to investigate the relationship between the dependent and independent variables. Variables associated with the dependent variables in the bivariate logistic regression analysis were subjected to multivariate analysis to control for potential confounders (p < 0.25). Finally, a p < 0.05 was used to measure statistical significance. The diagnostic agreement between the RDT and microscopy test results was evaluated using Cohen’s kappa coefficient.

## Results

### Socio-demographic characteristics of participants

A total of 573 out of 576 participants were included in the survey, with a response rate of 99.5%. In terms of gender distribution, 351 (61.3%) of the participants were males. Adults 15 years of age and above accounted for 285 (49.7%) while school-aged children (5 to 14 years old) and children under 5 years old accounted for 231 (40.3%) and 57 (9.9%), respectively. In general, 182 (31.8%) of participants did not have a formal education, while the remaining attended from elementary to bachelor’s degree level.

Regarding the housing condition, 405 (70.7%) of the houses were built with wood and mud and 508 (88.7%) of houses had eave at the ceiling and on the wall. Among the surveyed houses, only 101 (14.8%) households utilized nets to control vectors (Table 1). Nevertheless, none of the surveyed houses had been IRS-sprayed. IRS was not applied in the area for more than 3 years, according to data from the district health office.

**Table 1:** Socio demographic characteristics of participants at Boricha district in Sidama region, Ethiopia (April to May 2021)

Variable	Frequency	Percentage
Sex		
Male	351	61.3
Female	222	38.7
Age in year		
< 5	57	9.9
5–14	231	40.3
≥ 15	285	49.7
Marital status		
Married	242	42.2
Single	25	4.4
Divorced	1	0.2
Widowed	12	2.1
Age below 18 years	293	51.1
Educational status		
No formal education	182	31.8
Elementary school	339	59.2
Secondary school and above	52	9.1
Occupation		
Farmer	100	17.5
House wife	114	19.9
Merchant	16	2.8
Government/non-governmental organization employee	18	3.1
Student	325	56.7
House with window		
Yes	247	43.1
No	326	56.9
House with Eave		
Yes	508	88.7
No	65	11.3
Wall of the house		
Wood without mud	105	18.3
Wood with mud	405	70.7
Cement	63	11.0
Family size		
≤ 5	382	66.7
≥ 6	191	33.3
Insecticide-treated net (ITN) utilization		
Yes	101	17.6
No	472	82.4
Travel history		
Yes	29	5.1
No	544	94.9

### Prevalence of asymptomatic malaria infection

The detection of *Plasmodium* infection varied by diagnostic methods: microscopy and RDT. Of the 573 asymptomatic participants tested for malaria, 6.1% (95% Confidence Interval (CI) 4.3–8.4) were confirmed to be malaria positive by RDT and 4.0% (95% CI 2.6–6.0) were positive for malaria by microscopy. There was a substantial agreement between RDT and microscopy test results (kappa value, k = 0.67, p < 0.001 [34].

**Fig. 2:**
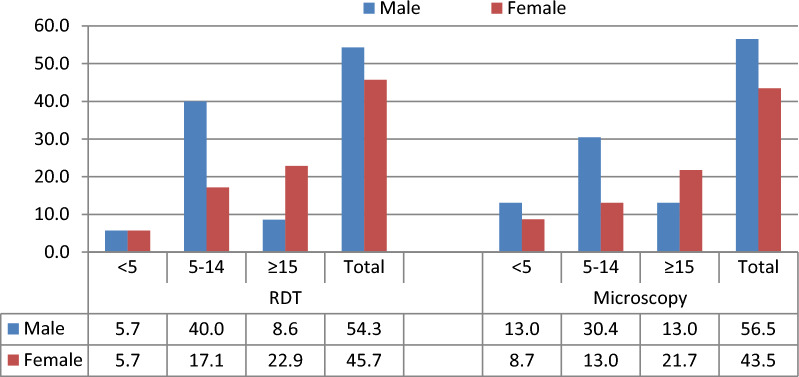
Proportion of asymptomatic malaria infection by age and sex in Boricha district, Sidama region, Ethiopia (April to May 2021)

Regardless of the diagnostic methods, males were generally more affected than females (54.3 vs. 45.7% by RDT and 56.5 vs. 43.5 by microscopy). Children in the high school age range (5–14) were more affected than children under five and adults aged 15 and above, as well (Fig. 2).

**Fig. 3:**
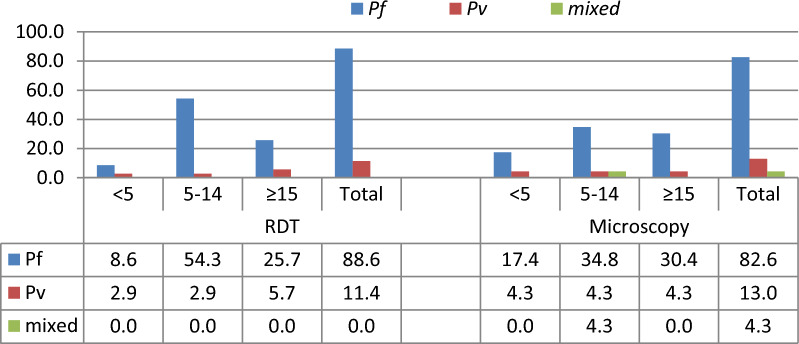
Distribution of *Plasmodium* species by age among asymptomatic malaria cases in Boricha district, Sidama region, Ethiopia (April to May 2021)

**Fig. 4:**
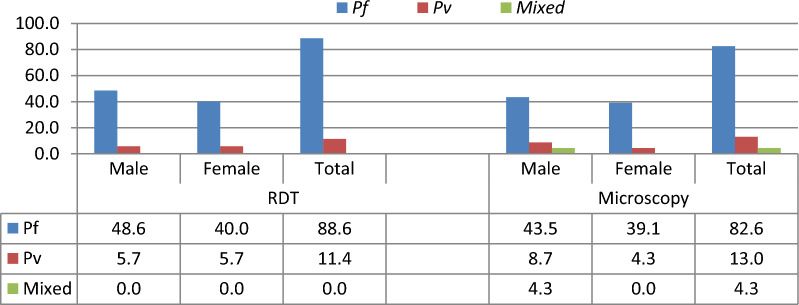
Distribution of *Plasmodium* species by sex among asymptomatic malaria cases in Boricha district, Sidama region, Ethiopia (April to May 2021)

In terms of species distribution, *P. falciparum* was the predominant as compared to *P. vivax* in all age groups and both sexes irrespective of the diagnostic tools (Figs. 3 and 4). As a consequence, *P. falciparum* and *P. vivax* accounted for 88.6% and 11.4% of RDT-confirmed cases, respectively, whereas *P. falciparum*, *P. vivax*, and mixed infections accounted for 82.6%, 13.0%, and 4.3% by microscopy confirmed cases.

The occurrence of malaria varied among different kebeles. Hence, it was 22.4% (13 cases out of 58 tested) in Sadamo Chala, 18.8% (3 cases out of 16 tested) in Harira Badalicha, 14.8% (8 cases out of 54 tested) in Balela, 5.75% (4 cases out of 70 tested) in Worancha Wacho, 3.9% (2 cases out of 51 tested) in Konsore Chafe, 2.2% (4 cases out of 185 tested) in Fulasa Aldada, 0.9% (1 case out of 115 tested) in Korangoge, and 0% (0 cases out of 24 tested) in Shondolo Liwo. The overall prevalence across all kebeles was 6.1% (35 cases out of 573 tested).

### Risk factors for asymptomatic malaria infection

The results of bivariate analysis showed that, age, ITN utilization, the availability of an eave and a window, previous travel experiences, educational status, and occupation were associated with the prevalence of asymptomatic malaria infection. All variables that were found to be significant during the bivariate analysis were then fitted to multivariate logistic regression in order to control the confounding variables (Table 2).

**Table 2:** Results of bivariate and multivariate logistic regression of associated factors with asymptomatic malaria infection, Boricha district, Sidama region, Ethiopia (April to May 2021)

Variable	Malaria lab result		Bivariate analysis		Multivariate analysis	
	Positive	Negative	COR (95% CI)	p-value	AOR (95% CI)	p-value
Age in year						
< 5	4	53	1.88 (0.58–6.13)	0.295	1.57 (0.46–5.39)	0.48
5–14	20	211	2.36 (1.10–5.04)	0.026	2.42 (1.08–5.40)	0.03*
≥ 15	11	274	1	0.08	1	0.10
ITN utilization						
Yes	1	100	1		1	
No	34	438	7.76 (1.05–57.38)	0.045	8.41 (1.09–65.08)	0.04*
Eave available						
Yes	34	474	4.59 (0.62–34.11)	0.136	6.41 (0.81–50.92)	0.08
No	1	64	1			
Window						
Yes	20	227	1.83 (0.91–3.65)	0.087	2.11 (1.02–4.36)	0.04*
No	15	311	1		1	
Travel history						
Yes	6	23	4.63 (1.75–12.26)	0.002	6.85 (2.32–20.26)	0.00*
No	29	515	1		1	
Educational status						
No formal education	11	171	3.28 (0.41–26.02)	0.26	2.32 (0.25–21.93)	0.46
Elementary school	23	316	3.71 (0.49–28.09)	0.20	2.54 (0.28–22.79)	0.41
Secondary and above	1	51	1	0.44		0.71

*p-value < 0.05

The multivariate analysis revealed that travel history, Insecticide-treated net utilization, presence of window, and age were significantly associated risk factors for malaria infection. Compared to the group of 15 years and above, the odd of contracting malaria among under 5 year age and 5–14 year age group was (AOR = 1.57, 95% CI 0.46–5.39) and (AOR = 2.42, 95% CI 1.08–5.40), respectively. The risk of contracting malaria was approximately 8.4 times higher in people who did not use ITNs compared to in those who did, (AOR = 8.41; 95% CI 1.09–65.08). Living in a home with window had also increased a risk of contracting malaria by 2.1 times compared to a home without a window (AOR = 2.11, 95% CI 1.02–4.36). Additionally, participants with the travel history to surrounding districts and towns had seven (times (AOR = 6.85, 95% CI 2.32–20.26) at higher risk of contracting the disease than those who had not (Table 2).

## Discussion

Ethiopia made remarkable gains in the fight against malaria, and it is currently on a promising path to elimination [4, 5]. In this regard, the country has already met the GTS 2020 targets of a 40% reduction in malaria morbidity and mortality [3]. Therefore, the elimination goal could most likely be achieved if the progresses obtained so far is maintained and the existing preventive interventions intensified [7, 35]. While appreciating the progress made thus far, it is crucial to understand the role that asymptomatic malaria plays in the journey of elimination [18, 36], and this was the focus of the current study.

According to the current study, the prevalence of asymptomatic malaria infection was 6.1%. This result was comparable to the pooled prevalence of asymptomatic malaria (6.7%), which was determined by a thorough review and meta-analysis of studies carried out in Ethiopia [26]. In addition, a comparable finding was reported in Ethiopia and elsewhere [37–39]. In addition, compared to findings from other areas of the country [40–42], asymptomatic malaria infection was more prevalent in the current study area.

On the other hand, compared to the current study, a higher prevalence of asymptomatic malaria infection was reported in the country [20, 21, 23, 24, 42, 43] and elsewhere [44, 45]. These variations could be attributed to several factors, including the group of the study population, geographical areas the study covered, sampling techniques, and the season of the study period as well. In this regard, while the current study included the entire population, others cited above focused on specific populations, such as pregnant women [24], school children [44], and seasonal migrant workers who travel for farm activities [23]. In addition, while the current study was carried out in a single area, the studies conducted elsewhere [20, 42, 43] covered a wide range of areas. Furthermore, unlike the current study, the other study [44] employed a repeated cross-sectional study design.

In addition, the current study was conducted during a low-transmission season, from April to May [7], while studies elsewhere in the country [23, 42] were carried out during the major malaria transmission seasons, from September to December [7]. This suggests that if the study had been conducted during the high transmission season, the prevalence of asymptomatic malaria infection would have been higher than the current finding.

In general, a recent study shows that malaria was persistently expanding in the community at an unacceptable prevalence, especially in light of the elimination target. Therefore, relying solely on passive case detection and management at the health facilities level might not be sufficient to meet the elimination goal. As a result, in addition to passive case detection, active and reactive case detection at the community level could prevent such silent transmission and eventually eliminating the disease. Additionally, it is strongly recommended to take into account mass testing and treatment activities; successful initiatives elsewhere [46, 47] to fight against the disease.

Attributable risk factors for malaria transmission, including socio-demographic factors, housing characteristics and the utilization of vector control interventions were also investigated in the current study. Regarding this, age of the participants, housing structure, utilization of preventive interventions, and travel history were identified risk factors for the infection. In this regard, the current study shows that children under the age of 15 years were more affected than adults aged 15 years and above. Particularly, children in the age group 5 to 14 years were more affected and responsible for a significant portion of the cases identified. Comparable reports were also reported in the country and elsewhere [48–51]. On the other hand, studies in Ethiopia and elsewhere reported that adults were more affected than children [31, 52].

The findings, therefore, suggested that the main reservoir of asymptomatic malaria infection in the study area was school-aged (5–14 years old) children. Similarly, studies from several countries found that school-aged children were the primary reservoir of asymptomatic infection. As a result, in order to control the infections and prevent further transmission in the population, interventions should be enhanced, with emphasis on these specific age groups [9, 53–55].

Study findings elsewhere also reported a high prevalence of malaria among children in the school-age group [53, 56]. This could be due to the insufficient attention given to the impact of the disease on this particular age group. As a result, there has been a lack of proper recognition and prioritization of the importance of addressing the disease in school-age children. This, in turn, has led to inadequate implementation of preventive strategies. Additionally, they have a lower likelihood of receiving timely detection and immediate treatment for the disease. The situation could not only increase their susceptibility to the disease but also make them a potential reservoir of the malaria parasite.

The current study also found a significant association between ITNs utilization and asymptomatic malaria infection. In this regard, those who did not use ITN had 8.41 times higher likelihood of acquiring malaria than those who did. Similarly, several studies reported that using an ITN reduces the risk of malaria infection [21, 57–59]. On top of this, the study also found that indoor residual spraying was not practiced for the last 6 months prior to the study period. Therefore, this could also be a possible reason for the increase the prevalence of the asymptomatic malaria in the community. Therefore, the study setting in particular and the nation as a whole should intensively utilize the two highly recommended vector control interventions, ITN and IRS [2], to attain the ultimate goal of elimination.

Several studies in Ethiopia and elsewhere reported that malaria transmission has a strong association with the housing characteristics of the community [60–65]. Consequently, the impact of house structure on the transmission of malaria was investigated in this study. However, the current study found that only the presence of windows in the houses had a strong association with asymptomatic malaria infection in the study area. Similarly, opening windows after retiring to sleep was associated with malaria transmission in South Africa [61].

Participants with confirmed malaria in the present study travelled to surrounding districts and towns in search of grazing lands, while some of them were also traders and others were students attending schools in surrounding districts and towns where malaria was more common. Similarly, several surveys conducted across the country and abroad have reported supporting results [34, 57, 66–68].

Thus, the study gave evidence that regardless of the distance travelled, residents’ movement from their homes to other areas were risk factors for malaria infection. Therefore, in general, the finding implies that malaria is not an area-specific problem confined to a single area, indicating elimination in a specific region depends on the disease status in neighboring areas. Therefore, considering the stratification of the malarious areas and thus identifying more affected areas is very crucial for the equitable distribution of prevention strategies to realize an elimination goal.

Regarding the *Plasmodium* species distribution, *P. falciparum was* a predominant species across all age groups and sexes as well. This finding is comparable with the studies conducted within the country and elsewhere [34, 57, 69]. In the study conducted in the same area before, *P. falciparum* was reported to be a predominant parasite species where it accounted for 56.3% while, *P, vivax* and mixed infections was 38.4%, and 5.2%, respectively [31]. Therefore, both species were important in the district, the intensive application of preventive interventions against both is still extremely important to achieve the elimination pathway. However, according to the current study, the share was much higher than before. This could be due to the fact that *P, vivax* had a lower parasite density than *P. falciparum*, which could lead to a reduced detection [43, 70, 71].

The recent finding has major implications for considering the risk of undiagnosed infection reservoirs in the context of anticipated elimination. In another sense, it implies that attention should be made to the disease’s silent spread rather than relying exclusively on the typical passive case identification at the facility level. As a result, it has the potential to notify all program stakeholders about the risks that lie ahead of the elimination track due to undiagnosed cases in the community, helping them to take appropriate action as soon as possible.

The finding has also significant implications for revisiting the surveillance, monitoring, and evaluation system of malaria elimination program. This is due to the fact that progress on the elimination path has been reported more frequently from health facilities through a passive surveillance system. As such, relying simply on such data may conceal the true picture of the disease at both the local and national levels. Thus, enhancing the integration of passive, active, and reactive case detection is highly recommended in order to identify and treat all cases and thereby prevent further transmission [1, 2, 72, 73].

The study has certain limitation. It used only conventional diagnostic methods, such as RDT and microscopy, which are inadequate when compared to more advanced techniques. Moreover, the potential influence of *hrp2* and *hrp3* gene deletions, which could affect test results, was not taken into account. Additionally, the study lacked detailed information on parasitaemia level or parasite density. Furthermore, it was conducted during the minor transmission season and focused exclusively on a certain kebeles in the district.

Therefore, it is highly recommended further to conduct an extensive study utilizing molecular diagnostic techniques to gain more precise understanding of disease prevalence in the community. Additionally, taking into account seasonal and spatial variations, including both rural and urban settings is highly recommended for the development and implementation of evidence-based interventions tailored to specific areas.

The current study excluded individuals who have had malaria in the past 3 months. However, this approach could overlook the possibility of reinfection and relapse cases, even when individuals do not exhibit symptoms. Therefore, it is highly recommended to include individuals who have been diagnosed with malaria and have completed a full treatment course following national guidelines in future studies. Finally, a comprehensive and extensive entomological study is essential to comprehensively understand the role of mosquitoes in the on-going transmission of diseases in the study area.

## Conclusion

The results of the study revealed that there is high prevalence of asymptomatic malaria infection in the study area. Thus, strengthening malaria diagnosis both at health facilities and community level, along with intensive implementation of vector control strategies could highly support reducing the disease transmission in the community and ultimately support the elimination goal. In addition, the study indicated that school aged children were the main reservoirs of the asymptomatic malaria infection. Therefore, devising well-targeted age specific interventions could incredibly block further transmission of the disease in the community.

## Data Availability

All data underlying the findings are available from corresponding authors on reasonable request.

## Abbreviations

WHO: World Health Organization
GTS: Global technical strategy
ITNs: Insecticide-treated bed nets
IRS: Indoor residual spraying
SPSS: Statistical Package for the Social Science
RDT: Rapid diagnostic test
BF: Blood films
HAD: Health development army

## References

[1] WHO (2017). A framework for malaria elimination.

[2] WHO (2015). Global technical strategy for malaria 2016–2030.

[3] WHO (2021). World malaria report 2021.

[4] PMI, Ethiopia malaria operational plan FY. 2022. https://www.pmi.gov. Addis Ababa, Ethiopia: Ministry of Health; 2022.

[5] FMoH (2021). Health sector transformation plan II.

[6] Gari T, Lindtjorn B (2018). Reshaping the vector control strategy for malaria elimination in Ethiopia in the context of current evidence and new tools: opportunities and challenges. Malar J.

[7] FMoH (2020). National malaria elimination strategic plan 2021–2025.

[8] Dhiman S (2019). Are malaria elimination efforts on right track? An analysis of gains achieved and challenges ahead. Infect Dis Poverty.

[9] Nkumama IN, O’Meara WP, Osier FHA (2017). Changes in malaria epidemiology in Africa and new challenges for elimination. Trends Parasitol.

[10] Mebratie AD, Nega A, Gage A, Mariam DH, Eshetu MK, Arsenault C (2022). Effect of the COVID-19 pandemic on health service utilization across regions of Ethiopia: an interrupted time series analysis of health information system data from 2019–2020. PLoS Glob Public Health.

[11] Shuka Z, Mebratie A, Alemu G, Rieger M, Bedi AS (2022). Use of healthcare services during the COVID-19 pandemic in urban Ethiopia: evidence from retrospective health facility survey data. BMJ Open.

[12] The PMI VectorLink Project (2021). *Anopheles stephensi* in Ethiopia: potential impact and mitigation.

[13] Balkew M, Mumba P, Dengela D, Yohannes G, Getachew D, Yared S (2020). Geographical distribution of *Anopheles stephensi* in eastern Ethiopia. Parasit Vectors.

[14] FMoH (2017). Surveillance, monitoring and evaluation manual for malaria elimination in Ethiopia.

[15] Bousema T, Okell L, Felger I, Drakeley C (2014). Asymptomatic malaria infections: detectability, transmissibility and public health relevance. Nat Rev Microbiol.

[16] Galatas B, Bassat Q, Mayor A (2016). Malaria parasites in the asymptomatic: looking for the hay in the haystack. Trends Parasitol.

[17] Andolina C, Rek JC, Briggs J, Okoth J, Musiime A, Ramjith J (2021). Sources of persistent malaria transmission in a setting with effective malaria control in eastern Uganda: a longitudinal, observational cohort study. Lancet Infect Dis.

[18] Lindblade KA, Steinhardt L, Samuels A, Kachur SP, Slutsker L (2013). The silent threat: asymptomatic parasitemia and malaria transmission. Expert Rev Anti Infect.

[19] WHO (2014). From malaria control to malaria elimination: a manual for elimination scenario planning.

[20] Mengesha E, Zerefa MD (2022). Asymptomatic malaria and nurturing factors in lowlands of Ethiopia: a community based cross-sectional study. PLoS Glob Public Health.

[21] Minwuyelet A, Eshetu T, Milikit D, Aschale Y (2020). Prevalence and risk factors of asymptomatic *Plasmodium* infection in Gondar Zuria District, Northwest Ethiopia. Infect Drug Resist.

[22] Goshu EM, Zerefa MD, Tola HH (2022). Occurrence of asymptomatic malaria infection and living conditions in the lowlands of Ethiopia: a community-based cross-sectional study. Infect Dis Poverty.

[23] Tilaye T, Tessema B, Alemu K (2022). High asymptomatic malaria among seasonal migrant workers departing to home from malaria endemic areas in northwest Ethiopia. Malar J.

[24] Nega D, Dana D, Tefera T, Eshetu T (2015). Prevalence and predictors of asymptomatic malaria parasitemia among pregnant women in the rural surroundings of Arbaminch Town, South Ethiopia. PLoS ONE.

[25] Tilahun A, Yimer M, Gelaye W, Tegegne B (2020). Prevalence of asymptomatic *Plasmodium* species infection and associated factors among pregnant women attending antenatal care at Fendeka town health facilities, Jawi District, North west Ethiopia: a cross-sectional study. PLoS ONE.

[26] Tamiru A, Tolossa T, Regasa B, Mosisa G (2022). Prevalence of asymptomatic malaria and associated factors in Ethiopia: systematic review and meta-analysis. SAGE Open Med.

[27] SNNPR. Annual performance report. Hawassa, Ethiopia: Finance and Economic Development Bureau; 2018.

[28] Sidama Zone. Annual performance report. Hawassa, Ethiopia: Agriculture Department; 2018.

[29] Sidama Zone. Annual performance report. Hawassa, Ethiopia: Health Department; 2018.

[30] RHB. Regional malaria report. Hawassa, Ethiopia: Sidama Regional Health Bureau; 2022.

[31] Dabaro D, Birhanu Z, Yewhalaw D (2020). Analysis of trends of malaria from 2010 to 2017 in Boricha District, Southern Ethiopia. Malar J.

[32] WHO (2009). Malaria microscopy quality assurance. Manual—version 1.

[33] FMoH (2018). National malaria guidelines.

[34] Amare A, Eshetu T, Lemma W (2022). Dry-season transmission and determinants of *Plasmodium* infections in Jawi district, northwest Ethiopia. Malar J.

[35] Bugssa G, Tedla K (2020). Feasibility of malaria elimination in Ethiopia. Ethiop J Health Sci.

[36] Drakeley C, Gonçalves B, Okell L, Slater H (2018). Understanding the importance of asymptomatic and low-density infections for malaria elimination.

[37] Abebaw A, Aschale Y, Kebede T, Hailu A (2020). The prevalence of symptomatic and asymptomatic malaria and its associated factors in Debre Elias district communities, Northwest Ethiopia. Malar J.

[38] Nzobo BJ, Ngasala BE, Kihamia CM (2015). Prevalence of asymptomatic malaria infection and use of different malaria control measures among primary school children in Morogoro Municipality, Tanzania. Malar J.

[39] Benié EMA, Silué KD, Ding XC, Yeo I, Assamoi JB, Tuo K (2022). Accuracy of a rapid diagnosis test, microscopy and loop-mediated isothermal amplification in the detection of asymptomatic *Plasmodium* infections in Korhogo, Northern Côte d’Ivoire. Malar J.

[40] Zerdo Z, Bastiaens H, Anthierens S, Massebo F, Masne M, Biresaw G (2021). Prevalence and associated risk factors of asymptomatic malaria and anaemia among school-aged children in Dara Mallo and Uba Debretsehay districts: results from baseline cluster randomized trial. Malar J.

[41] Bansil P, Yeshiwondim AK, Guinovart C, Serda B, Scott C, Tesfay BH (2018). Malaria case investigation with reactive focal testing and treatment: operational feasibility and lessons learned from low and moderate transmission areas in Amhara Region, Ethiopia. Malar J.

[42] Nega D, Abera A, Gidey B, Mekasha S, Abebe A, Dillu D (2021). Baseline malaria prevalence at the targeted pre-elimination districts in Ethiopia. BMC Public Health.

[43] Hailemeskel E, Tebeje SK, Behaksra SW, Shumie G, Shitaye G, Keffale M (2021). The epidemiology and detectability of asymptomatic *Plasmodium vivax* and *Plasmodium falciparum* infections in low, moderate and high transmission settings in Ethiopia. Malar J.

[44] Mensah BA, Myers-Hansen JL, Amoako EO, Opoku M, Abuaku BK, Ghansah A (2021). Prevalence and risk factors associated with asymptomatic malaria among school children: repeated cross-sectional surveys of school children in two ecological zones in Ghana. BMC Public Health..

[45] Agaba BB, Rugera SP, Mpirirwe R, Atekat M, Okubal S, Masereka K (2022). Asymptomatic malaria infection, associated factors and accuracy of diagnostic tests in a historically high transmission setting in Northern Uganda. Malar J.

[46] Singh A, Rajvanshi H, Singh MP, Bhandari S, Nisar S, Poriya R (2022). Mass screening and treatment (MSaT) for identifying and treating asymptomatic cases of malaria-malaria elimination demonstration project (MEDP), Mandla, Madhya Pradesh. Malar J.

[47] Ndong IC, Okyere D, Enos JY, Mensah BA, Nyarko A, Abuaku B (2019). Prevalence of asymptomatic malaria parasitaemia following mass testing and treatment in Pakro sub-district of Ghana. BMC Public Health.

[48] Loha E, Lindtjørn B (2012). Predictors of *Plasmodium falciparum* malaria incidence in Chano Mille, South Ethiopia: a longitudinal study. Am J Trop Med Hyg.

[49] Pinchoff J, Chaponda M, Shields TM, Sichivula J, Muleba M, Mulenga M (2016). Individual and household level risk factors associated with malaria in Nchelenge District, a region with perennial transmission: a serial cross-sectional study from 2012 to 2015. PLoS ONE.

[50] Gbalégba CGN, Ba H, Silué KD, Ba O, Tia E, Chouaibou M (2018). Distribution of *Plasmodium* spp. infection in asymptomatic carriers in perennial and low seasonal malaria transmission settings in West Africa. Infect Dis Poverty.

[51] Earland DE, Bibe AF, Novela A, Ferrão J, Searle KM (2022). *Plasmodium falciparum* community prevalence and health-seeking behaviours in rural Sussundenga District, Mozambique. Malar J.

[52] Tefera S, Bekele T, Getahun K, Negash A, Ketema T (2022). The changing malaria trend and control efforts in Oromia special zone, Amhara regional state, North-East Ethiopia. Malar J.

[53] Nankabirwa J, Brooker SJ, Clarke SE, Fernando D, Gitonga CW, Schellenberg D (2014). Malaria in school-age children in Africa: an increasingly important challenge. Trop Med Int Health..

[54] Kleinschmidt I, Sharp B (2001). Patterns in age-speci®c malaria incidence in a population exposed to low levels of malaria transmission intensity. Trop Med Int Health..

[55] Griffin JT, Ferguson NM, Ghani AC (2014). Estimates of the changing age-burden of *Plasmodium falciparum* malaria disease in sub-saharan Africa. Nat Commun.

[56] Walldorf JA, Cohee LM, Coalson JE, Bauleni A, Nkanaunena K, Kapito-Tembo A (2015). School-age children are a reservoir of malaria infection in Malawi. PLoS ONE.

[57] Fekadu M, Yenit MK, Lakew AM (2018). The prevalence of asymptomatic malaria parasitemia and associated factors among adults in Dembia district, northwest Ethiopia, 2017. Arch Public Health.

[58] Adugna F, Wale M, Nibret E (2022). Prevalence of malaria and its risk factors in Lake Tana and surrounding areas, northwest Ethiopia. Malar J.

[59] Yadav K, Dhiman S, Rabha B, Saikia P, Veer V (2014). Socio-economic determinants for malaria transmission risk in an endemic primary health centre in Assam, India. Infect Dis Poverty.

[60] Wanzirah H, Tusting LS, Arinaitwe E, Katureebe A, Maxwell K, Rek J (2016). Mind the gap: house structure and the risk of Malaria in Uganda. PLoS One.

[61] Coleman M, Coleman M, Mabaso MLH, Mabuza AM, Kok G, Coetzee M (2010). Household and microeconomic factors associated with malaria in Mpumalanga, South Africa. Trans R Soc Trop Med Hyg.

[62] Tesfay K, Assefa B, Addisu A (2019). Malaria outbreak investigation in Tanquae Abergelle district, Tigray region of Ethiopia: a case–control study. BMC Res.

[63] Dlamini N, Hsiang MS, Ntshalintshali N, Pindolia D, Allen R, Nhlabathi N (2017). Low-quality housing is associated with increased risk of malaria infection: a national population-based study from the low transmission setting of Swaziland. Open Forum Infect Dis.

[64] Bradley J, Rehman AM, Schwabe C, Vargas D, Monti F, Ela C (2013). Reduced prevalence of malaria infection in children living in houses with window screening or closed eaves on Bioko Island, Equatorial Guinea. PLoS ONE.

[65] Essendi WM, Vardo-Zalik AM, Lo E, Machani MG, Zhou G, Githeko AK (2019). Epidemiological risk factors for clinical malaria infection in the highlands of western Kenya. Malar J..

[66] Yukich JO, Taylor C, Eisele TP, Reithinger R, Nauhassenay H, Berhane Y (2013). Travel history and malaria infection risk in a low-transmission setting in Ethiopia: a case control study. Malar J.

[67] Sarkar R, Kessler A, Mawkhlieng B, Sullivan SA, Wilson ML, Carlton JM (2021). Household and individual level risk factors associated with declining malaria incidence in Meghalaya, India: implications for malaria elimination in low-endemic settings. Malar J.

[68] Haile M, Lemma H, Weldu Y (2017). Population movement as a risk factor for malaria infection in high-altitude villages of Tahtay–Maychew District, Tigray, northern Ethiopia: a case–control study. Am J Trop Med Hyg.

[69] Tesfahunegn A, Berhe G, Gebregziabher E (2019). Risk factors associated with malaria outbreak in Laelay Adyabo district northern Ethiopia, 2017: case-control study design. BMC Public Health.

[70] Koepfli C, Robinson LJ, Rarau P, Salib M, Sambale N, Wampfler R (2015). Blood-stage parasitaemia and age determine *Plasmodium falciparum* and *P. vivax* gametocytaemia in Papua New Guinea. PLoS ONE.

[71] Ahmed S, Reithinger R, Kaptoge SK, Ngondi JM (2020). Travel is a key risk factor for malaria transmission in pre-elimination settings in sub-Saharan Africa: a review of the literature and meta-analysis. Am J Trop Med Hyg.

[72] The malERA Group on Consultative on Monitoring, evaluation, and Surveillance (2011). A research agenda for malaria eradication: monitoring, evaluation, and surveillance. PLoS Med..

[73] Ohrt C, Roberts KW, Sturrock HJW, Wegbreit J, Lee BY, Gosling RD (2015). Information systems to support surveillance for malaria elimination. Am J Trop Med Hyg.

